# Restoring Teeth with an Advanced Lithium Disilicate Ceramic: A Case Report and 1-Year Follow-Up

**DOI:** 10.1155/2022/6872542

**Published:** 2022-09-16

**Authors:** Felicitas Hölken, Helmut Dietrich

**Affiliations:** Department of Prosthodontics and Materials Science, University Medical Center of University of Mainz, Augustusplatz 2, 55131 Mainz, Germany

## Abstract

Advancements in materials science and bonding protocols as well as new manufacturing methods foster the development of novel ceramic materials to meet the increased demands for highly aesthetic, biocompatible, and long-lasting restorations in fixed prosthodontics. This case report highlights the minimally invasive rehabilitation with a new advanced lithium disilicate (ALD) ceramic block. It is reinforced with virgilite crystals in managing esthetic demand of patient besides having a high flexural strength. According to the manufacturer, the material provides a biaxial strength measured at >700 MPa and improved optical properties. The remarkable speed sintering time of approx. 4.5 minutes makes processing very fast. Time efficiency, predictability, and economically interesting treatment options are of great importance in current dentistry and can be well implemented in CAD/CAM dentistry. The newly introduced ALD ceramic for the “Chairside Economical Restoration of Esthetic Ceramics”/“CEramic REConstruction” (CEREC) system produces an esthetically pleasing and clinically excellent restoration. The shorter processing time combined with high flexural strength will optimize the chairside workflow. New treatment indication options for lithium disilicate ceramics will expand. Although more evidence from long-term clinical studies is needed to verify the clinical performance and manufacturer recommendations regarding indication, preparation and cementation must be followed very strictly. In the present case report, restorations were indicated for seven posterior teeth, which were prepared, scanned, designed with CEREC-Primescan SW 5.1.3, and fabricated with MCX5. The monolithic restorations were placed adhesively. The rehabilitation with the ALD blocks resulted in an aesthetically pleasing, functional outcome that improved overall treatment time and increased patient and practitioner satisfaction, which remained stable over a one-year follow-up period.

## 1. Introduction

Everybody is talking about digitalization today. It impacts not only social life but also the way dentistry is performed. The advent of computerized technologies in restorative dentistry has led to an enormous change for dentists and dental technicians. Indirect restorations can now be made in dental practices (chairside), laboratories, or even production centers [1, 2]. The use of digitally generated data sets, computer-aided design, and numerical control (NC) technology for processing silicate and oxide ceramics allows us to work with new, industrially prefabricated and almost defect-free restorative materials [1]. Dental ceramics are known for their high esthetics and biocompatibility. Because of these properties and the patients' demand for materials that are as lifelike as possible, ceramics are widely used in present-day dentistry. While in the past almost exclusively manual techniques, such as layering or pressing, were used to produce ceramic restorations, computer-aided design and computer-aided manufacturing (CAD/CAM) technology now help us to process completely new materials, which are unfit for manual processing. In the 1990s, the first oxide ceramic crowns and bridges were made, thanks to new computer-aided grinding and milling systems and polycrystalline crystals reinforced with 5.35% by weight of yttrium oxide [3]. As a viable alternative to metal-based restorations, featuring a high esthetic potential and excellent biocompatibility, these ceramics have become very popular and have replaced metals in a variety of indications [4–6]. After numerous improvements, we can now distinguish between five generations of modern zirconia restoratives. Users should be aware of the indications and limitations of these different ceramics. Just like silicate ceramics, they differ greatly in their optical and mechanical properties [5]. Technique sensitivity in the processing steps, chipping of veneering materials, or even fracturing of zirconia frameworks gave rise to criticism in the past [4, 6–10].

In recent years, there has been a trend toward the use of monolithic restorations to avoid any chipping of veneering ceramics on oxide ceramic frameworks [5, 11, 12].

Monolithic crowns can be made of silicate or oxide ceramics. Ultimately, the users of CEREC systems were the ones who demanded industrially prefabricated ceramics with clearly defined properties, suitable for chairside grinding in one appointment. These industrial ceramic blocks are characterized by a controlled, uniform structural quality. This is important when using ceramics, because every pore, every irregularity, may cause cracking and lead to the failure of a restoration. Because of the benefits of adhesive bonding, short production times and high esthetics, numerous CEREC users prefer glass ceramic blocks. High success and survival rates of chairside fabricated restorations have been described [3, 10, 13–15]. Lithium disilicate (LS2) ceramics are the most frequently used materials in chairside processing and is particularly proven in terms of esthetics and strength and therefore widely accepted and used (e.g., CEREC Tessera, Dentsply Sirona, Charlotte, USA; IPS e.max CAD, Ivoclar Vivadent, Schaan, Liechtenstein) [15, 16]. A relatively high glass content also allows users to adhesively bond restorations after etching with hydrofluoric acid, and thanks to their higher strength of more than 350 MPa (3-point flexural strength), as compared to other glass ceramics, LS2 ceramics requires less tooth structure reduction in the preparation step. This opens the doors to defect-oriented, nonretentive preparation designs. In this combination, it is suitable for a large variety of indications, including single-tooth crowns, partial crowns, veneers, inlays, onlays, or implant-supported restorations. Even according to the manufacturers' information, 3-unit fixed partial dentures (FPDs) up to the first premolar can be fabricated (e.g., IPS e.max CAD, Ivoclar Vivadent, Schaan, Liechtenstein). A prerequisite for this is compliance with the minimum material thickness of 1.0 mm and the demanding adhesive bonding [17, 18]. Following the trend toward monolithic restorations [5], LS2 ceramics can even be used in esthetically demanding cases without additional veneering, thanks to its translucency [15, 19].

**Figure 1:**
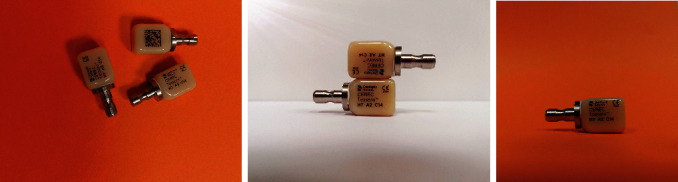
CEREC tessera blocks.

The following case report demonstrates the fabrication of seven monolithic single-tooth restorations by using a novel advanced lithium disilicate (ALD) ceramic (CEREC Tessera, Dentsply Sirona, Charlotte, USA) fabricated with the CEREC system (Primescan and MC X5, Dentsply Sirona, Charlotte, USA) (Figure 1). The material is a tooth-colored block but needs a mandatory firing to reach its final strength. This ALD ceramic features a special microstructure. According to the manufacturer, it consists of LS2 and virgilite, i.e., lithium aluminum silicate, embedded in a glass matrix enriched with zirconia. During firing, more virgilite crystals are formed. On the one hand, the rod-shaped LS2 crystals create high tensile strength, counteracting crack propagation, and on the other hand, the small virgilite crystals formed during firing substantially contribute to the high biaxial flexural strength of >700 MPa by increasing precompression stress.

## 2. Case History

**Figure 2:**
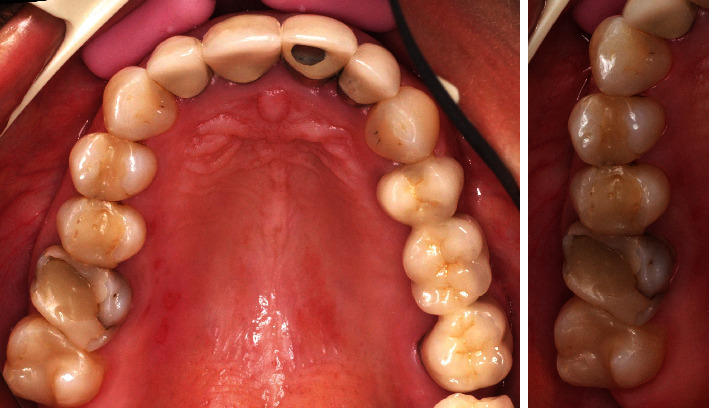
Initial situation of the maxilla: teeth 16-14 insufficient composite restorations; tooth 16 endodontically treated.

**Figure 3:**
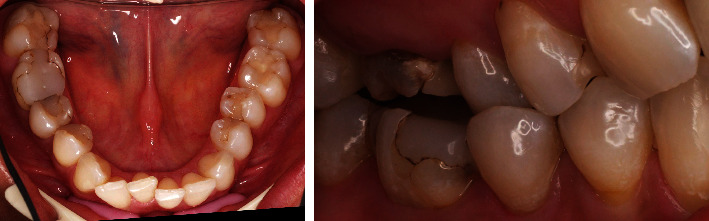
Initial situation in the mandible: teeth 44-47 insufficient composite restorations; tooth 45, 46 endodontically treated; lateral view: open edge-to-edge bite in the region of teeth 16, 46 and a cross bite in the region of teeth 15, 45.

A patient presented in the Prosthodontic Department of the Dental Clinic of the University Mainz, Germany. Teeth 16-14 and 44-47 had been restored with large composite fillings. Parts of these restorations had fractured shortly after placement and had been repeatedly repaired. Teeth 16, 45, and 46 had also been endodontically treated several years ago. The X-ray diagnostics revealed a sufficient endodontic treatment ad-apex without apical findings and the patient was pain-free. Teeth 16 and 46 showed minor discolorations of the tooth structure. The other vital teeth showed insufficient restoration margins and carious lesions (Figures 2 and 3). The patient chewed only on the left side, for fear of losing her fillings again, and wished to finally receive durable restorations. In addition to an improvement in her functional situation, esthetics mattered to the patient. An open edge-to-edge bite in the region of teeth 16, 46 and a cross bite in the region of teeth 15, 45 were diagnosed. No additional temporomandibular disorders were found.

### 2.1. Material Selection

Seven restorations were made using the CEREC system. To simplify the complexity of the bite, the fourth quadrant (44-47) was treated first. Depending on defect size, teeth 47 and 44 received inlays, tooth 45 received an onlay, and tooth 46 received a partial crown. Adhesively bonded restorations have frequently proven to be a successful alternative to traditional crowning [20]. For a tooth affected by an extensive intracoronal lesion, like tooth 46 in this case, a crown is often preferred as the treatment of choice. However, a crown preparation will usually weaken the residual tooth structure even further when there is such a large defect [21]. An adhesively bonded partial ceramic crown can be an alternative in this situation. It is less invasive, because of its defect-oriented preparation design [22–24]. To preserve as much tooth structure as possible, a material which, thanks to high flexural strength, helps to minimize the height of the restoration while still meeting high esthetic requirements was selected. After completing the restorations of the mandibular teeth, the maxillary teeth (14–16) were treated. Due to the root canal treatment and the loss of substance, the tooth 16 received a partial crown. With regard to the defect size and as much preservation of tooth structure as possible, tooth 15 received an onlay, and tooth 14 an inlay; all restorations were adhesively bonded.

### 2.2. Preparation and Digital Workflow

**Figure 4:**
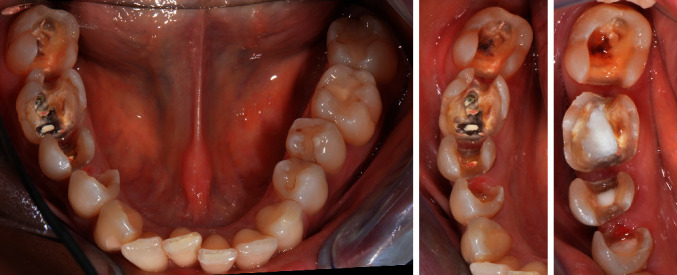
Cavity preparations and bases in the mandible.

Shade selection using a conventional shade guide (Vitapan classic, Vita Zahnfabrik, Bad Säckingen, Germany) was performed. Following the removal of the existing composites from teeth 44-47 and caries excavation, cavity bases were placed to cover the root canal fillings and cavity floors (Clearfil DC core plus, Dentin, Kuraray Noritake Dental Inc., Tokyo, Japan) (Figure 4). Subsequent preparations had to meet the material-specific requirements of CAD/CAM milled ceramics [25]. For defect-oriented partial crown preparation, minimum wall thicknesses recommended by the manufacturer (1.5 mm for occlusal and 1.0 axial walls) were strictly adhered to and internal line angles were rounded (4562.314, Komet Dental, Lemgo, Germany). The occlusal design from the partial crowns (teeth 16 and 46) were prepared with a rhombic (8899.314.027, Komet Dental, Lemgo, Germany) and a bud-shaped instrument (8368.314.016, Komet Dental, Lemgo, Germany) [25, 26]. The inlay preparation (1.0 mm occlusal minimal width and height) was free of undercuts, all line angles were rounded, a taper of approx. 6°–10° relative to the occlusal surface was prepared, and the margins were not located in occlusal contact points (8863.204.012 and 4562.314, Komet Dental, Lemgo, Germany). Sharp line angles should be avoided, especially when using CAM inlays, because they may create problems during production and placement [25].

**Figure 5:**
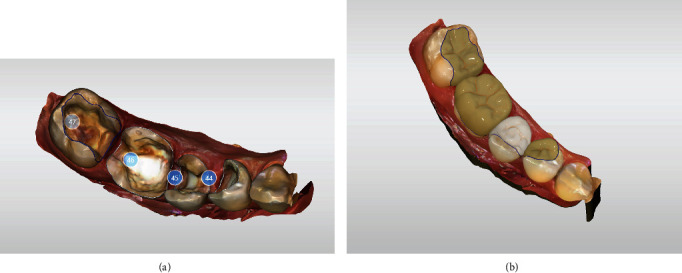
Digital design for teeth 44, 45, 46, and 47; CEREC SW 5.1.3.

**Figure 6:**
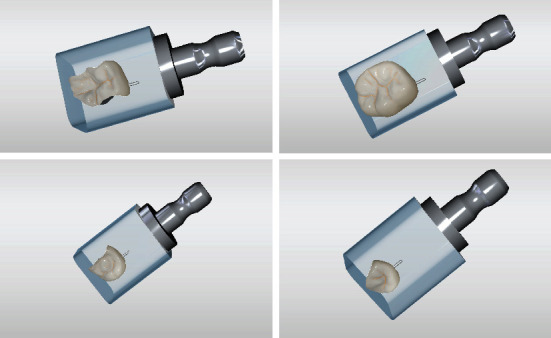
Restorations designed for teeth 47, 46, 45, and 44, ready for grinding.

The quadrant, the opposite jaw, and a lateral bite registration were intraorally scanned (CEREC Primescan AC SW 5.1.3, Dentsply Sirona, Charlotte, USA). The automatically determined restoration proposals have improved immensely with new software updates that deliver reliable restorations that hardly need to be modified. Due to the number of restorations, the decision was made in this case to set the model axis, enter the preparation margin, and define the insertion axis oneself. The software used in the biogeneric individual mode presented a restoration design for the missing occlusal surfaces. Various tools can be used to easily customize this design (Figures 5 and 6). A modification of the restoration design was only necessary by adjusting the occlusal and proximal contacts. Digital scanning was followed by the placement of temporary restorations. The chairside restorations were not placed in this appointment, considering the scope of this work, the cross bite, and the need to individually stain the ceramic.

**Figure 7:**
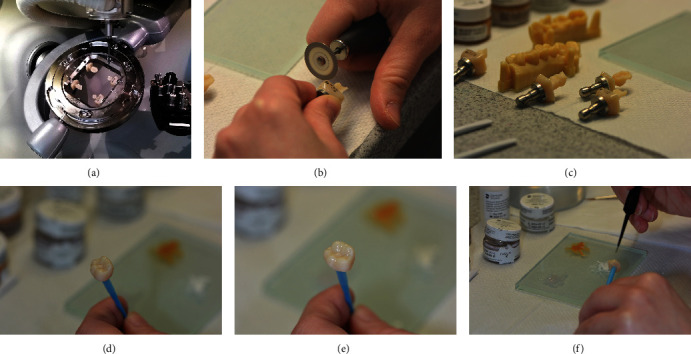
(a) Technical workflow: grinding restorations with MC X5; (b) & (c) handling preparations (d)–(f) staining and glazing restorations.

**Figure 8:**
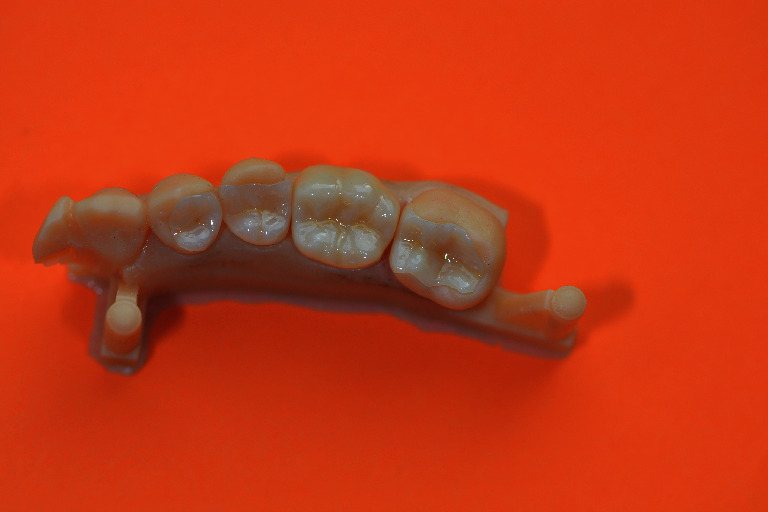
Finalization of the restorations 47, 46, 45, and 44 on a printed model.

**Figure 9:**
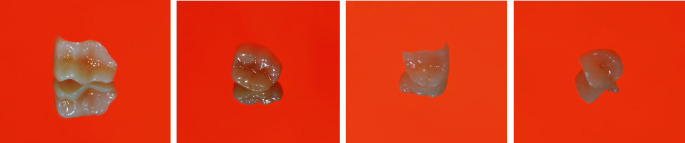
Final restorations of the teeth 47, 46, 45, and 44.

After initial shade determination, CEREC Tessera blocks in size C14 (18 × 14 × 12 mm) were selected in shade MT A2 for teeth 46 and 47, and in shade HT A2 for the other teeth. The blocks come in a variety of shades and in two translucencies, and differ in fluorescence depending on the shade. HT (High Translucency) blocks are suitable for the production of inlays, onlays, or anterior restorations, which should be highly translucent. Medium translucency (MT) blocks are more opaque and therefore preferably used for posterior restorations masking discolored tooth structure or cast post and cores. After grinding in the CEREC MC X5 production unit, the retention pin of the crown was removed, and the connecting area was finished with a fine-grid diamond instrument (Figure 7). The restorations were checked for accuracy of fit and occlusal contacts on a printed model (SolFlex, W2P, Vienna, Austria) and adjusted where necessary (Figure 8). Then the restorations were individually stained and glazed for furnace firing (DS Body Stain S1, DS Incisal Stain S1, DS Mahogany, Dentsply Sirona, Charlotte, USA). The stains should be thoroughly mixed with a non-metallic spatula and diluted on a glass slab as desired before application. The restorations should be clean and free of grease. The restorations were placed on a firing tray with a firing pad (DeguDent, Hanau-Wolfgang, Germany) and fired at 760°C (Figure 9). The restorations should be positioned in the center to ensure they are exposed to the correct temperature. The firing is indispensable to achieving the high final strength of this ceramic; firing for individual characterization with stains is optional. Polishing alone will not lead to high final strength, in contrast to other (glass) ceramics (e.g., IPS e.max CAD, Ivoclar Vivadent, Schaan, Liechtenstein; Celtra duo, Dentsply Sirona, Charlotte, USA). Any conventional ceramic furnace will allow users to easily set the right firing parameters. Using a SpeedFire furnace (Dentsply Sirona, Charlotte, USA), however, the firing time of CEREC Tessera restorations can be reduced to 4.5 minutes. Prior to speed firing in this furnace, only spray glaze needs to be applied to the ceramic. Finally, the restoration was high-shine polished with a diamond polishing paste (Fegupol 8059, Feguramed, Buchen, Germany) (Figure 9.

### 2.3. Placement

**Figure 10:**
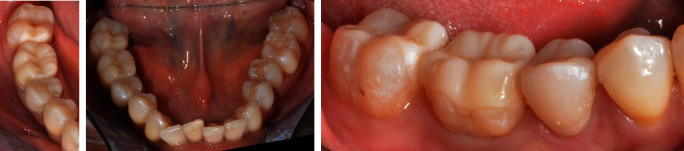
Restorations placed on teeth 44, 45, 46, and 47.

**Figure 11:**
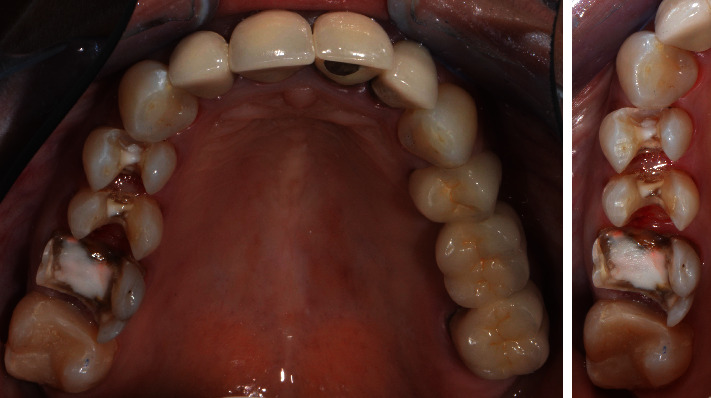
Cavity preparations and bases in the maxilla.

**Figure 12:**
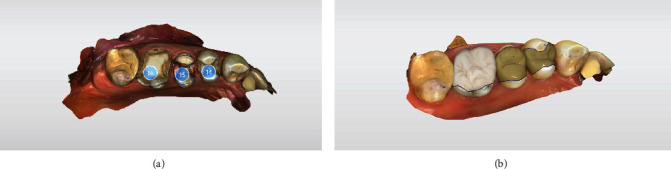
Digital design for teeth 16, 15, and 14; CEREC SW 5.1.3.

**Figure 13:**
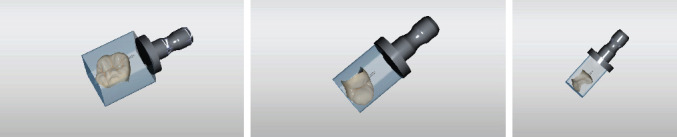
Restorations designed for teeth16, 15, and 14, ready for grinding.

**Figure 14:**
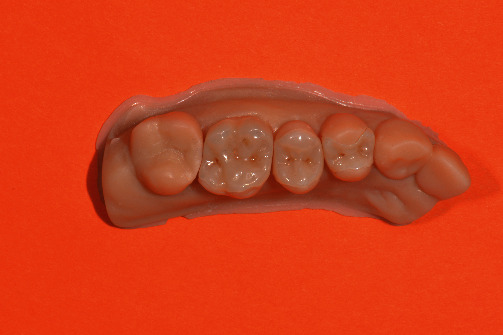
Finalization of the restorations 16, 15 and 14 on a printed model.

**Figure 15:**
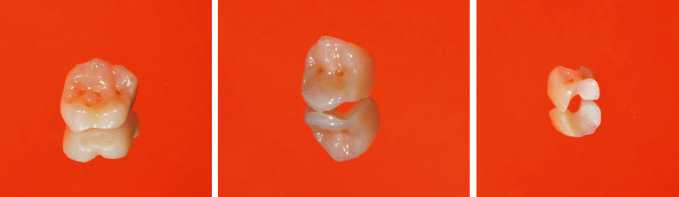
Final restorations for teeth 16, 15 and 14.

**Figure 16:**
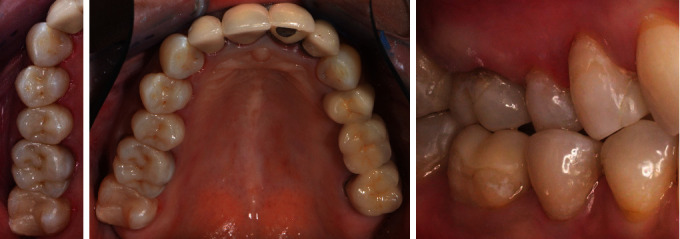
Restorations placed on teeth 16, 15 and 14 with final lateral view.

The restorations were checked for marginal and interproximal fit. They all fitted accurately without any looseness or gaps. Then the order in which the restorations would be placed was determined. The ceramic was cleaned with alcohol, and the inner surfaces were etched with 5% hydrofluoric acid gel (IPS Ceramic Etching Gel, Ivoclar Vivadent, Schaan, Liechtenstein). After etching for 30 seconds, the surfaces were cleaned, first in a water beaker and then with air/water spray. The dried restoration surfaces were coated with a silane coupling agent (Calibra silane, Dentsply Sirona, Charlotte, USA) for 60 seconds, and then surplus was removed by strong air stream. The enamel surfaces were selectively etched with 37% phosphoric acid gel, rinsed with water spray, and carefully air-dried. Then a universal adhesive (Prime & Bond active, Dentsply Sirona, Charlotte, USA) was applied to the dry tooth structure for 20 seconds and light-cured after solvent evaporation. The restorations were luted using adhesive resin cement (Calibra Ceram, Dentsply Sirona, Charlotte, USA). Gross excess cement was removed with foam pellets, and interproximal areas were cleaned with dental floss. This was followed by tack-curing to easily remove the remaining excess cement in a gel state with a scaler. Then, each surface was light-cured for at least 20 seconds, and the margins were polished if necessary, followed by polishing with a three-step ceramic polisher set (New Technology Instruments, P30032A, Kahla, Germany). Static and dynamic occlusions were checked. The restoration proved to be a good color match with the adjacent teeth (Figure 10). After placing the mandibular restorations, the maxillary teeth (16-14) were prepared, and a digital impression was taken, in the same appointment (Figures 11 and 12). The restorations were produced as described above (Figures 13, 14, and 15) and placed in another appointment (Figure 16). At the follow-up visit, the patient was very satisfied with the esthetic results of the treatment and dared to bite normally again. To prevent excessive stressing of the restorations, a relaxation splint to be worn at night was made. The esthetics, functional occlusion, and gingival tissue remained stable over a follow-up period of 6 months and 1 year. No signs of fractures within the restorations were observed.

### 2.4. Discussion

CAD/CAM is a promising technology and has been employed in the chairside fabrication of all-ceramic restorations [27, 28]. The advance of this science and a high demand for metal-free restorations have led to rapid advancements in processing technologies and the development of newer restorative materials [15]. With continuous improvements in intraoral scanning technology, new software updates and material innovations, this technique is becoming increasingly widespread [29–32].

Due to their high strength combined with great translucency, LS2 ceramic monolithic blocks have become the material of choice for chairside dentistry [15, 16].

This case report describes the fabrication of seven monolithic restorations with a novel ALD ceramic. On account of the number of restorations, the restorations were milled with the CEREC MCX5. Taking the path of chairside production, the CEREC Primemill in the superfast mode would have reduced the grinding time compared to previous chairside milling processes (CEREC MCXL) [18, 28, 33].

Due to the constant development of the software (CEREC SW 5, Dentsply Sirona, Charlotte, USA) the design process has become simpler and more intuitive [31, 34, 35]. The current software offers practical design proposals that mainly only need minor adjustments at the occlusal and proximal contact points [17], which leads to a significant reduction in time [18].

As one major advantage of the digital workflow, the time needed for occlusal and internal adjustments is shorter than for restorations fabricated in the conventional workflow [27, 31, 34].

The optional speed-sintering process reduces the fabrication time significantly. While the influence of the speed-sintering process on the mechanical and optical properties of zirconia ceramics has been investigated in several in vitro studies [36–39], further studies of this relatively new processing technique on ALD ceramics would be necessary.

The high flexural strength of ALD ceramics present an advantage of this new material in comparison to other LS2 ceramics and might expand the range of indications for restorations exposed to high stress areas. Compared to zirconia ceramics the processing of ALD is easier, faster, and practicable for chairside use.

Flexural strength is a critical material property, evaluated in standardized tests. The value of >700 MPa indicated by the manufacturer was achieved in biaxial flexural tests. As there are different testing methods, such as three-point, four-point or biaxial flexural tests, measurements are difficult to compare [40]. The flexural strength of the ALD ceramic considerably exceeds that of other silicate ceramics. This is why it can be both adhesively bonded and conventionally cemented, the latter only if retentive preparation is carried out and the manufacturer's instructions have been followed (minimum wall thicknesses of full crowns for conventional cementation: occlusal and axial walls 1.5 mm each; for adhesive bonding: occlusal and axial walls 1.0 mm each for all indications except onlays, which need 1,5 mm occlusal thickness). And you can count on the material's strength even at wall thicknesses down to 1.0 mm, giving you greater flexibility in your restoration designs. A study on LS2 ceramics showed a significant increasing strength of the restoration when an adhesive cement is used rather than a conventional one [41]. Whereas the adhesive bond of glass-ceramics with low flexural strength (e.g., feldspar or leucite ceramics) is necessary for mechanical strength of the restoration [42]. In the present case report, the ALD ceramic was conditioned by an etching process with 5% hydrofluoric acid for 30 seconds. This pretreatment ensures a bonding strength comparable to the well examined LS2 ceramics in combination with dual curing composite cements and self-adhesive cements [43]. A recently published in vitro study showed that there is only a minor effect of cementation technique (adhesive resin, glass-ionomer cement, and hybrid glass-ionomer cement) of the ALD ceramic on durability during in vitro mastication and fracture force [44]. The same study showed a significantly lower wear of ALD and a tendency toward lower antagonist wear than LS2 [44]. Further long-term clinical trials are needed here to confirm this.

According to the manufacturer, the crystal sizes of 500–700 nm correspond to the wavelengths of visible light; this is intended to lead to an increased light diffusion effect that is independent of wavelengths. High light transmission and diffusion create a chameleon effect, so that restorations blend in with the natural tooth structure. The translucent properties of LS2 ceramics are a major advantage compared to zirconia ceramics. Also the ALD ceramics deliver the tooth-like esthetics of glass ceramics which can be confirmed in the present case report. The blocks are currently only available in monochromatic color and can be stained for individual characterization. Especially for the production of crowns and partial crowns a multilayer block with a prefabricated color gradient could facilitate the staining process and could be a further time-saving innovation.

An alternative treatment option for the case presented would have been the fabrication of restorations made from a high-strength glass-ceramics (e.g., IPS e.max CAD, Ivoclar Vivadent, Schaan, Liechtenstein). Due to the remaining walls of the endodontically treated teeth, a post build-up was not necessary [45]. Nevertheless, it is a limitation of these new materials that data from clinical studies are missing. Therefore, the manufacturer's instructions must be carefully observed.

## 3. Conclusion

Chairside restorations are becoming more and more important in dental practice. Industrially prefabricated ceramic blocks allow dentists to produce monolithic restorations in one appointment with optional finalization in a laboratory. The ALD ceramic combines a high biaxial flexural strength of >700 MPa and esthetically pleasing results. Beside to the wide range of indications for chairside fabrication, the high flexural strength also allows restorations to be made in load-bearing areas. In addition to the benefits of ease of use and good material properties, time efficiency plays a crucial role in dental practice. The time saving speed-sintering process of approx. 4.5 minutes makes processing very fast. Therefore, this ceramic is an interesting material for monolithic restorations produced in a digital workflow. Results from additional clinical studies are required to validate the positive results from these initial clinical experiences.

## Data Availability

The data that support the findings of this study are available from the author, upon reasonable request.

## Abbreviations

ALD: Advanced lithium disilicate
CEREC: Chairside economical restoration of Esthetic ceramics/CEramic REConstruction
NC: Numerical control
CAD/CAM: Computer-aided design/computer-aided manufacturing
LS2: Lithium disilicate
FPDs: Fixed partial dentures
HT: High translucency
MT: Medium translucency
CAD/CAM: Computer-aided design and computer-aided manufacturing.
